# A Case of TAFRO (Thrombocytopenia, Anasarca, Fever or Fibrosis of the Bone Marrow, Renal Dysfunction, and Organomegaly): Exploring the Idiopathic Variant of Multicentric Castleman Disease

**DOI:** 10.7759/cureus.107204

**Published:** 2026-04-16

**Authors:** Thanmayi Parasu, Niyanshi Gaddam, Taheea R Ahmed, Aasrith Parasu, Nithya Palanisamy

**Affiliations:** 1 School of Medicine, University of Texas Medical Branch at Galveston, Galveston, USA; 2 Hematology and Oncology, Baylor Scott and White Medical Center - McKinney, McKinney, USA; 3 School of Health Sciences, University of North Texas, Denton, USA

**Keywords:** anasarca, cytokine therapy, immunosuppression, inguinal lymphadenopathy, multicentric castleman disease (mcd), myelofibrosis, renal dysfunction, systemic inflammation, tafro syndrome

## Abstract

Characterized by Thrombocytopenia, Anasarca, Fever or Fibrosis of the bone marrow, Renal dysfunction, and Organomegaly, TAFRO syndrome is a rare systemic inflammatory disorder often presenting with diffuse lymphadenopathy and characteristic lymph node histopathology. Given its rapid progression and overlapping presentation with other systemic diseases, early diagnosis and treatment are vital for improving patient outcomes. Here, we report the case of a 43-year-old male with a medical history of chronic kidney disease and heart failure who presented with progressive anasarca and respiratory distress. The composite of clinical, laboratory, and histopathologic findings met the diagnostic criteria for TAFRO syndrome. The patient was treated with high-dose corticosteroids and anti-interleukin-6 therapy, leading to improvement of his symptoms. This case underscores the importance of integrating clinical features with histopathologic findings for early recognition and prompt initiation of immunosuppressive and anti-cytokine therapy to optimize the clinical outcomes of patients with TAFRO syndrome.

## Introduction

Castleman disease represents a heterogeneous group of lymphoproliferative disorders characterized by systemic inflammation, lymph node hypervascularity, and dysregulated cytokine signaling, particularly involving interleukin-6 (IL-6). It is broadly classified into unicentric Castleman disease, in which a single lymph node region is affected, and symptoms are often mild or absent, and multicentric Castleman disease (MCD), a more severe systemic variant associated with generalized lymphadenopathy, constitutional symptoms, cytopenias, and multi-organ dysfunction. MCD can occur in association with human herpesvirus-8 (HHV-8) infection or can be idiopathic (iMCD), the latter thought to arise from cytokine dysregulation without an identifiable infectious trigger.

Within the spectrum of iMCD is a distinct clinical subtype with Thrombocytopenia, Anasarca, Fever or Fibrosis of the bone marrow, Renal dysfunction, and Organomegaly known as TAFRO syndrome. TAFRO syndrome presents more acutely and severely than other forms of iMCD. Patients frequently demonstrate only mild lymphadenopathy despite profound systemic illness, and lymph node biopsies typically show features of atypical or mixed-type Castleman pathology. TAFRO syndrome is rare, with most published cases reported from Japan, although cases are now described in Western populations. Available series suggest a possible slight male predominance and a typical onset in middle age, although these findings remain uncertain due to small sample sizes. Notably, the two-year mortality rate in patients with TAFRO syndrome rises to 33.5%, whereas iMCD patients without TAFRO exhibit a two-year mortality rate of 0% [1-3].

TAFRO syndrome can be life-threatening, with rapid progression to multi-organ failure if not recognized promptly. Early diagnosis and aggressive therapy, which commonly includes corticosteroids, anti-IL-6 agents such as tocilizumab or siltuximab, and immunosuppressants including rituximab or cyclosporine, are critical for achieving remission. Since its first description in 2010, diagnostic criteria have continued to evolve, and consensus proposals were published in 2016.

Despite increasing recognition, significant knowledge gaps remain. The current literature consists mainly of small case reports and limited retrospective cohorts, leaving uncertainty regarding the full clinical spectrum, optimal diagnostic markers, therapeutic responsiveness, and prognostic indicators. Epidemiologic data also remain limited, and distinctions between TAFRO syndrome and other subtypes of iMCD are not well defined. The purpose of this work is to address these gaps by providing a comprehensive synthesis of current evidence, clarifying diagnostic considerations, and outlining emerging issues in the management of this rare entity [4,5].

Importantly, while TAFRO syndrome shares the underlying cytokine-driven pathophysiology of iMCD, it is distinguished by a more fulminant clinical course with disproportionate systemic inflammation, severe thrombocytopenia, marked anasarca, and acute renal failure, often requiring urgent escalation of immunosuppressive therapy compared with other iMCD subtypes.

## Case presentation

A 43-year-old male with a past medical history of hypertension, heart failure with reduced ejection fraction, chronic kidney disease stage 3a, and recurrent ascites requiring frequent paracentesis presented to the hospital with shortness of breath and anasarca.

On initial examination, the patient appeared distressed and edematous, with vital signs notable for hypotension (blood pressure: 85/55 mmHg), tachycardia (heart rate: 111 beats/minute), tachypnea (respiratory rate: 22 breaths/minute), and a temperature of 98.3°F (36.8°C). Physical examination revealed bibasilar crackles and abdominal ascites, with laboratory tests indicative of microcytic anemia, thrombocytopenia, hyponatremia, and a normal lactic acid level. Additional laboratory evaluation revealed acute kidney injury superimposed on chronic kidney disease, with a serum creatinine level of 6.4 mg/dL. The metabolic panel demonstrated a high anion gap metabolic acidosis, with an anion gap of 21, bicarbonate of 14 mmol/L, sodium of 135 mmol/L, and chloride of 100 mmol/L (Table 1).

**Table 1:** Admission metabolic and electrolyte panel. This table summarizes the patient’s admission laboratory values, demonstrating severe renal dysfunction with azotemia, hyperkalemia, and high anion gap metabolic acidosis consistent with acute kidney injury requiring renal replacement therapy.

Laboratory test	Result	Reference range
Sodium (Na)	135 mmol/L	135–145 mmol/L
Potassium (K)	6.0 mmol/L	3.5–5.1 mmol/L
Chloride (Cl)	100 mmol/L	98–107 mmol/L
Bicarbonate (HCO₃⁻)	14 mmol/L	22–28 mmol/L
Anion gap	21	8–12
Blood urea nitrogen (BUN)	93 mg/dL	7–20 mg/dL
Creatinine	6.4 mg/dL	0.7–1.3 mg/dL

Given the presence of anasarca, ascites, and renal dysfunction, chronic liver disease, including cirrhosis, was considered; however, there was no clinical, laboratory, or imaging evidence of hepatic failure to support this diagnosis. Additionally, the patient had no history of significant alcohol use or other risk factors for chronic liver disease.

**Figure 1:**
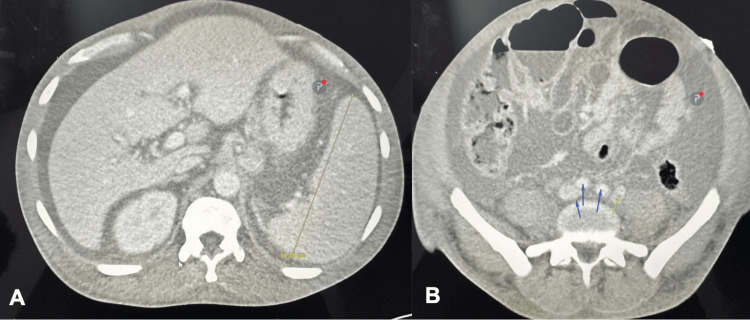
Axial CT scans of the abdomen displaying (A) hepatosplenomegaly, as demarked by the yellow line, and ascites. Imaging further reveals (B) retroperitoneal lymphadenopathy, highlighted by the arrows.

The patient was admitted to the intensive care unit, where continuous renal replacement therapy was initiated. CT imaging further supported the diagnosis by revealing hepatosplenomegaly, ascites, and extensive retroperitoneal and inguinal lymphadenopathy, radiologic findings consistent with TAFRO syndrome (Figures 1A, 1B).

Hematology/Oncology was consulted during admission, and the patient subsequently underwent a minimally invasive image-guided inguinal lymph node biopsy performed by interventional radiology for diagnostic evaluation of lymph node involvement. Following the procedure, the patient developed respiratory distress requiring endotracheal intubation. He was managed with intravenous diuretics for volume overload, including intravenous furosemide with dosing titrated to clinical response, and an isoproterenol infusion was initiated for intermittent bradycardia. Given the complexity of his condition, he was subsequently transferred to a tertiary care university medical center for further management. There, he was immediately extubated and weaned off the isoproterenol drip. Apart from intermittent episodes of ileus, the patient’s condition continued to gradually improve on the floor.

**Figure 2:**
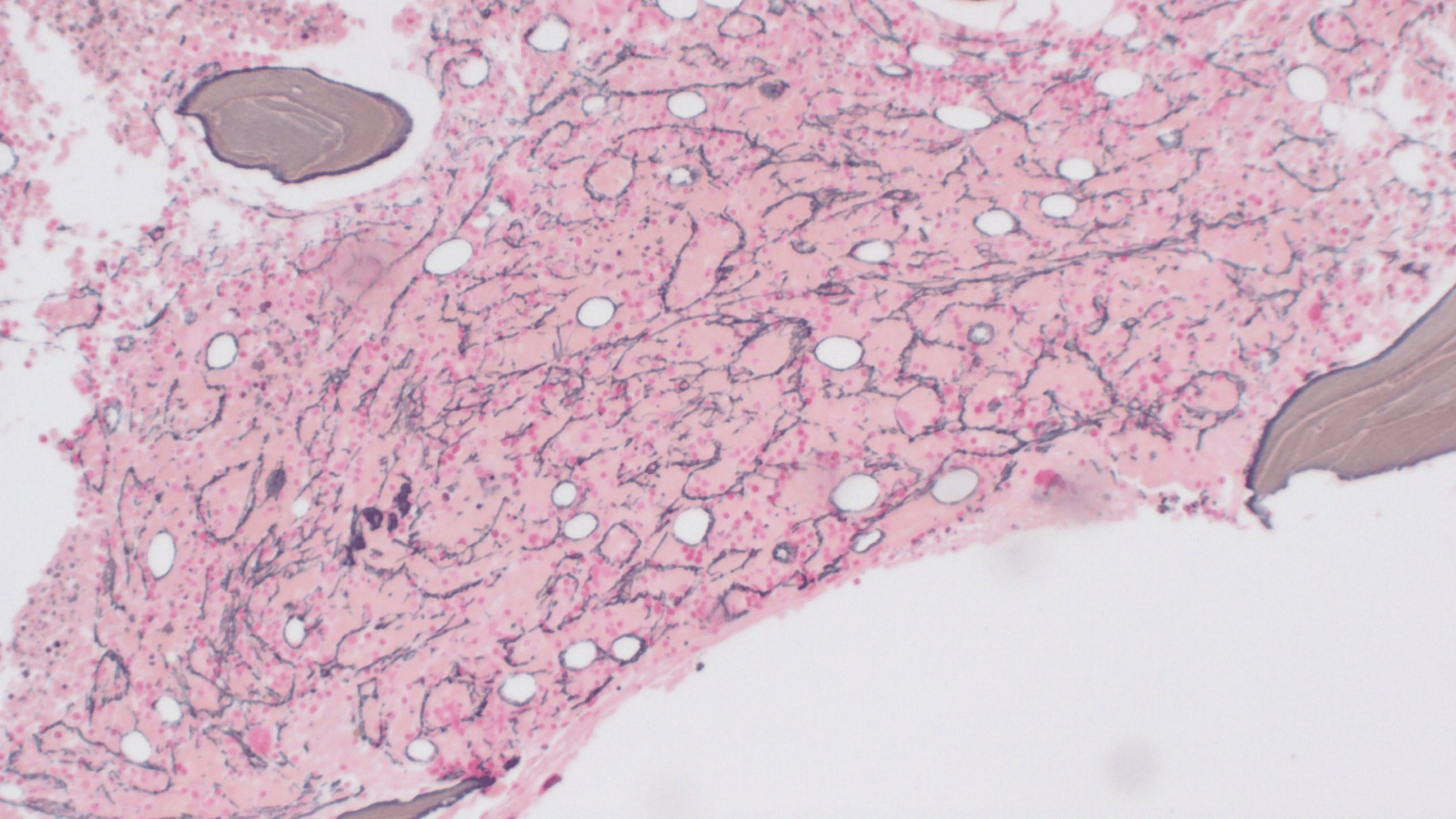
Section of the right groin lymph node showing many small atretic primary follicles and vascular proliferation.

Pathology from the inguinal canal lymph node resection revealed sections of the right groin lymph node showing many small atretic primary follicles, vascular proliferation, patent sinuses, and mild polyclonal plasmacytosis (Figure 2). No definitive morphologic or flow cytometric evidence indicated lymphoma. These reactive, Castleman-like changes in lymph nodes have often been described in autoimmune diseases due to the dysregulation of inflammatory mediators, such as nuclear factor-kB, IL-6, vascular endothelial growth factor, and JAK-STAT pathway proteins [6-8].

**Figure 3:**
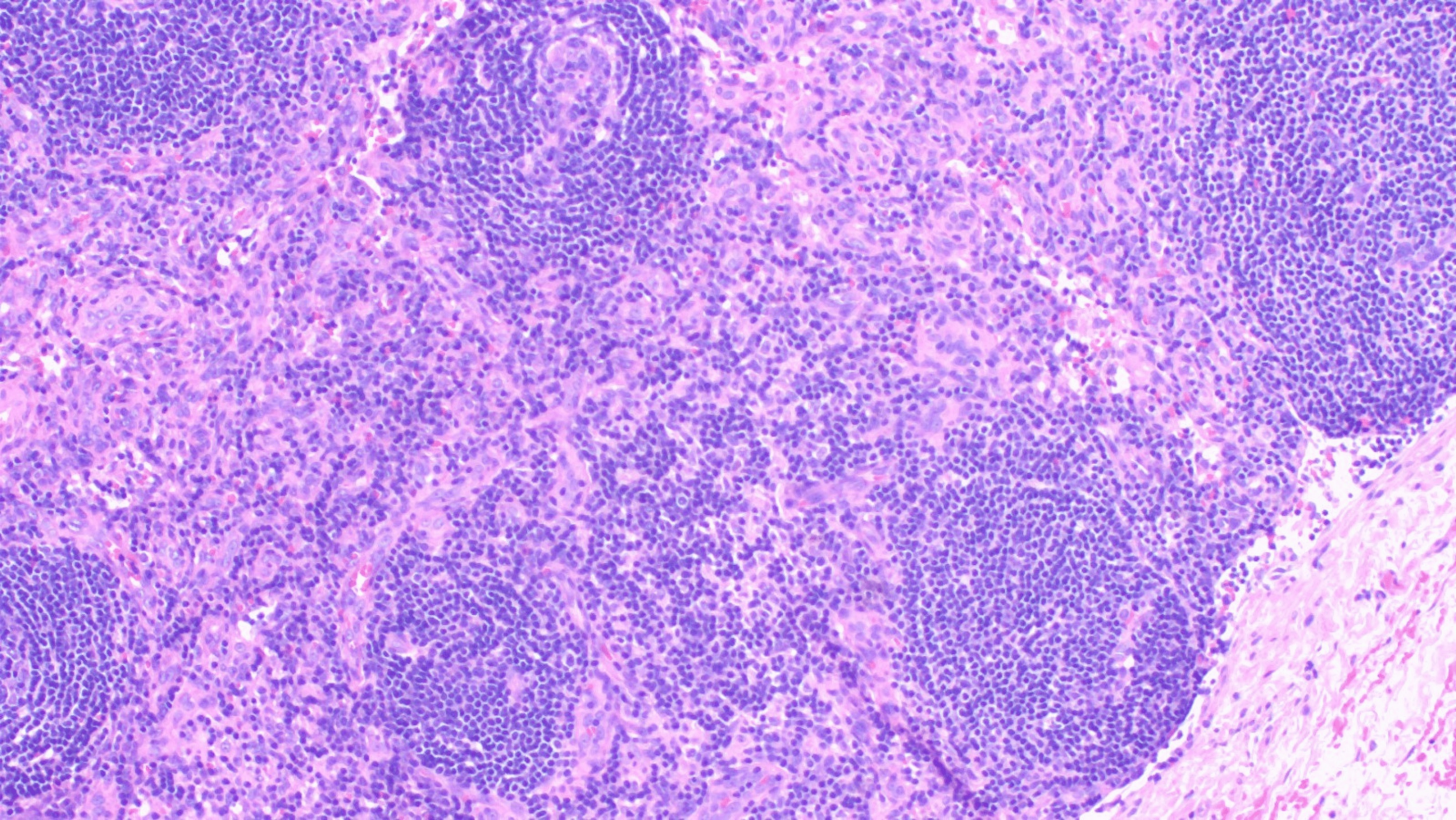
Patent sinuses and mild polyclonal plasmacytosis are observed, consistent with Castleman-like changes in lymph nodes.

Immunohistochemistry showed CD20 and PAX5 highlighted B cells predominating in primary follicles, while CD3 and CD5 highlighted T cells (Figure 3). CD10 and BCL6 identified rare germinal centers, which were appropriately negative for BCL2. CD35 highlighted many small follicular dendritic cell networks/intact follicles. MUM1 and CD138 showed a mild increase in plasma cells, which were polyclonal by kappa and lambda in situ hybridization studies. CD30 showed few scattered positive cells, and Ki67 indicated a low proliferation rate (5%). BCL1, C-MYC, pancytokeratin, EBER in situ hybridization, and HHV-8 were negative.

For further diagnostic evaluation, an Interventional Radiology (IR)-guided bone marrow biopsy of the right iliac crest revealed 75-80% cellular marrow with trilineage maturation, an increased number of megakaryocytes, and myeloid predominance. Iron stores were additionally elevated. There was minimal reticulin fibrosis (MF grade 0 to focal 1+ of 3). The peripheral blood showed hypersegmented neutrophils, marked normocytic anemia, and moderate thrombocytopenia. Cytogenetics revealed 46,XY. An autoimmune workup was performed and was unremarkable, including negative antinuclear antibody, antineutrophil cytoplasmic antibodies, SSA, and SSB, with normal complement levels (C3 and C4) and IgG; C-reactive protein was 1.95, and no evidence of an underlying autoimmune disorder was identified.

Based on the clinical presentation of thrombocytopenia, anasarca, myelofibrosis, renal failure, and lymphadenopathy, along with lymph node and bone marrow biopsy findings consistent with multicentric Castleman disease and TAFRO syndrome, high-dose corticosteroids and IL-6 blockade with siltuximab at 11 mg/kg every three weeks were initiated. Following treatment initiation, the patient demonstrated gradual clinical improvement. Anasarca and volume overload improved with diuresis and renal replacement therapy, and renal function showed partial recovery with decreasing creatinine levels over time, allowing transition off continuous renal replacement therapy. Hematologic parameters improved, with rising platelet count and hemoglobin levels during treatment. Organomegaly and inflammatory markers also showed interval improvement on follow-up imaging and laboratory testing. The patient completed five days of intravenous methylprednisolone 1 g daily, followed by prednisone 80 mg daily with a structured taper over three weeks, while continuing scheduled siltuximab therapy as maintenance immunotherapy.

## Discussion

MCD is a rare, systemic lymphoproliferative disorder characterized by widespread lymphadenopathy and systemic inflammation [3,6]. The TAFRO syndrome variant of MCD is particularly severe, manifesting with thrombocytopenia, anasarca, fever, renal dysfunction, and organomegaly. Diagnosis is based on clinical, laboratory, and histopathological findings, as seen in this case, where lymph node biopsy showed characteristic Castleman-like changes without evidence of lymphoma. Table 2 summarizes these diagnostic characteristics; a patient must meet all three of the major criteria and two out of the four minor criteria [5].

**Table 2:** Diagnostic features of TAFRO syndrome. TAFRO = Thrombocytopenia, Anasarca, Fever or Fibrosis of the bone marrow, Renal dysfunction, and Organomegaly

Category	Diagnostic feature
Major criteria	Anasarca (i.e., generalized edema, ascites)
	Thrombocytopenia (often <100 × 10⁹/L)
	Systemic inflammation (characterized by fever exceeding 37.5°C/elevated serum C-reactive protein)
Minor criteria	Renal insufficiency
	Organomegaly (i.e., liver, lymph nodes)
	Castleman-like features (seen majorly in lymph nodes)
	Reticulin myelofibrosis and/or an increased number of megakaryocytes in bone marrow
Exclusion criteria	Malignancy (especially lymphoma)
	POEMS (Polyneuropathy, Organomegaly, Endocrinopathy, Monoclonal gammopathy, and Skin changes) syndrome
	Infectious disorders
	Hepatic cirrhosis
	Thrombotic thrombocytopenic purpura (TTP)/Hemolytic uremic syndrome (HUS)
	Other autoimmune causes

TAFRO syndrome can present similarly to other systemic inflammatory conditions, including autoimmune diseases and hematological malignancies, making prompt diagnosis and treatment essential for improved outcomes. Treatment of this condition takes a more aggressive approach in comparison to Castleman disease due to its more rapidly progressing course. When promptly administered, higher dosages of immunosuppressive therapies with corticosteroids and agents targeting IL-6, such as tocilizumab, can reduce the systemic inflammation and lymphoproliferation associated with TAFRO syndrome [7].

## Conclusions

TAFRO syndrome is a distinct and rapidly progressive subtype of iMCD that requires a high level of clinical suspicion, particularly in patients who present with unexplained thrombocytopenia, anasarca, fever or fibrosis, renal dysfunction, and mild lymphadenopathy. This case highlights the importance of combining clinical, radiologic, and histopathologic findings to achieve timely diagnosis in a condition where delays can lead to significant morbidity and mortality. Early initiation of targeted therapy, including corticosteroids and IL-6 inhibition, can result in meaningful clinical improvement even in patients with underlying cardiac and renal comorbidities. As knowledge of TAFRO syndrome continues to grow, further research is needed to clarify its pathogenesis, refine diagnostic criteria, and develop evidence-based treatment approaches. Continued reporting of cases such as this one is essential to improving recognition, guiding management decisions, and, ultimately, enhancing outcomes for patients affected by this rare and challenging condition.
